# Reconsultation and Antimicrobial Treatment of Urinary Tract Infection in Male and Female Patients in General Practice

**DOI:** 10.3390/antibiotics5030031

**Published:** 2016-09-15

**Authors:** Meera Tandan, Sinead Duane, Martin Cormican, Andrew W. Murphy, Akke Vellinga

**Affiliations:** 1 Discipline of General Practice, School of Medicine, National University of Ireland Galway (NUIG), Galway, Ireland; sinead.s.duane@nuigalway.ie (S.D.); andrew.murphy@nuigalway.ie (A.W.M.); akke.vellinga@nuigalway.ie (A.V.); 2 Discipline of Bacteriology, School of Medicine, National University of Ireland Galway (NUIG), Galway, Ireland; martin.cormican@hse.ie; 3 Department of Medical Microbiology, University Hospital Galway (UHG), Galway, Ireland

**Keywords:** urinary tract infection, reconsultation, general practice, antimicrobial prescribing, treatment, culture test, male and female

## Abstract

Current antimicrobial prescribing guidelines indicate that male and female patients with urinary tract infections (UTIs) should be treated with same antimicrobials but for different durations. The aim of this study was to explore the differences in reconsultations and antimicrobial prescribing for UTI for both males and females. A total of 2557 adult suspected UTI patients participating in the Supporting the Improvement and Management of Prescribing for urinary tract infection (SIMPle) study from 30 general practices were analyzed. An antimicrobial was prescribed significantly more often to females (77%) than males (63%). Nitrofurantoin was prescribed more often for females and less often for males (58% vs. 41%), while fluoroquinolones were more often prescribed for males (11% vs. 3%). Overall, reconsultation was 1.4 times higher in females, and if the antimicrobial prescribed was not the recommended first-line (nitrofurantoin), reconsultation after empirical prescribing was significantly higher. However, the reconsultation was similar for males and females if the antimicrobial prescribed was first-line. When a urine culture was obtained, a positive culture was the most important predictor of reconsultation (Odds ratio 1.8 (95% CI 1.3–2.5)). This suggests, when prescribing empirically, that male and female UTI patients should initially be treated with first-line antimicrobials (nitrofurantoin) with different durations (50–100 mg four times daily for three days in females and seven days for males). However, the consideration of a culture test before prescribing antimicrobials may improve outcomes.

## 1. Introduction

Research on urinary tract infections (UTIs) has mainly been focused on women because of the higher incidence and prevalence in women compared to men [1,2]. Treatment recommendations for UTI differ for males and females, mainly regarding the duration of treatment. The Irish antimicrobial guidelines for treatment of UTI indicate the treatment of females and males with the same first-line antimicrobials (Nitrofurantoin and Trimethoprim) but for different durations (three days vs. seven days) [3]. However, the Scottish Intercollegiate Guideline Network (SIGN guidelines) UK indicate a 7 day first-line (Trimethoprim and Nitrofurantoin) treatment regimen for males with uncomplicated UTI, but antimicrobial treatment is not recommended for females [4]. One common conception in general practice is that males generally have complicated UTIs, and that therefore treatment recommendations for uncomplicated UTI in women are not appropriate for men. In an observational study of male veterans, treatment outcome (recurrence) was compared with the duration of treatment. The study showed that a longer duration of treatment (greater than seven days) was associated with increased late recurrence (30 days after the prior episode) [5]. They also found a link between the longer duration of treatment and the occurrence of *C. difficile* associated diarrhea in patients. Considering the risks associated with long courses of antimicrobials and no clinical benefit of the longer duration of treatment, Trautner suggests in her commentary for a more judicious use of antimicrobials for UTI in males [6]. A German observational study conducted in 2004 in 90 males concluded that UTI in males should not be treated empirically or based on dipstick results and clinical information. Their study showed that 60% of males had a positive culture even though half of them had low colony counts, and that the antibiotic prescribed to 36% of the males was not well targeted. The authors recommended to await urine culture results before a treatment decision is made [7]. A Dutch study of UTI in males reported that dipstick information in combination with clinical diagnosis was as accurate as the recommended care based on culture results [8]. The subsequent study comparing uropathogens and their resistance between male and female UTI patients similarly suggested that given the heterogeneous population of uropathogens causing UTI in males, empiric treatment should be avoided and treatment should be based on culture results [9]. Differences in treatment between these studies may be cultural or related to differences in the study populations and shows the need for more research to elucidate the epidemiology, diagnosis, and treatment of UTI in males. Also, in the wake of the global spread of antimicrobial resistance, limiting the use of antimicrobials is essential. 

In this study, we aim to describe the differences between males and females UTI patients attending to General Practices with respect to antimicrobial prescribing, and the frequency of reconsultation.

## 2. Results

A total of 3561 UTI consultations were recorded over a 15-month period, 372 (10.5%) males and 3187 (89.5%) females. There were 2557 (71.8%) index consultations (280/2557 males, 2275/2557 females and 2/2557 unknown) and 1004 (28.2%) additional consultations with a similar distribution of males (92/1004) and females (912/1004). The additional consultations were made within 30 days of the first visit (reconsultation) in the case of 512 (20%) of the index patients. An overview of the index consultation is presented in Table 1. Males were older than females and were more often patients holding a medical card.

### 2.1. Antimicrobial Prescribing

**Figure 1:**
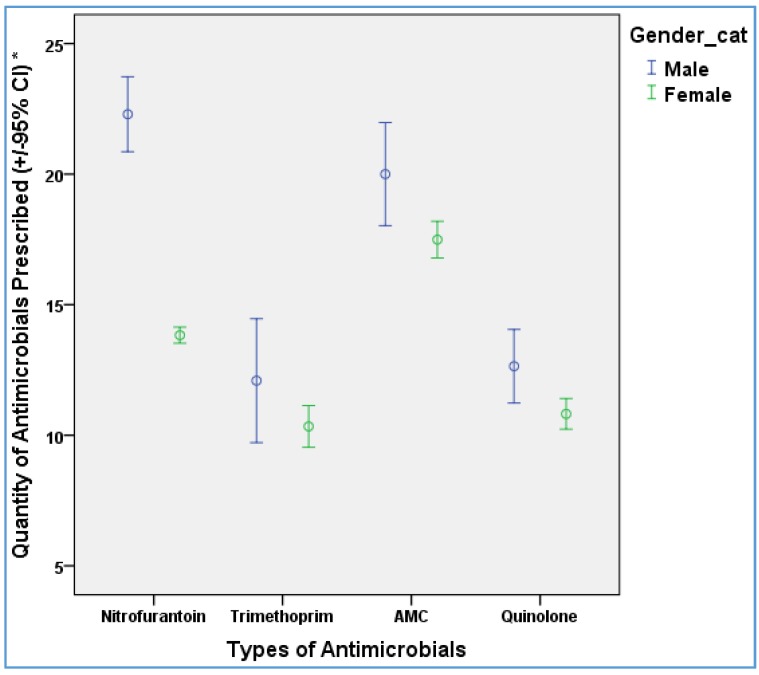
The types of antimicrobial prescribing based on the script quantity by gender. * The quantity of antimicrobial prescribing is measured as the dose dispensed, i.e., number of tablets prescribed.

Overall, an antimicrobial was prescribed to 75% of the patients at the index consultation and this was significantly higher for females (77%) compared to males (63% (Table 1)). When prescribed an antimicrobial, most received nitrofurantoin (75%) while 7% received trimethoprim and 5% received a fluoroquinolone (not shown in Table 1). Females were prescribed nitrofurantoin significantly more often (58% vs. 41%) while males received fluoroquinolones more often compared to females (11% vs. 3.1%). No significant differences were observed for the other antimicrobials. The duration of treatment, measured as the duration of the dose dispensed, was significantly higher for males compared to females (Table 1). The median duration of treatment for nitrofurantoin was significantly higher for males compared to females; seven days for males (median quantity 28/4 times daily (QDS)) and three days for females (median quantity 12, QDS) (Figure 1). There were no differences in the duration of fluoroquinolones and co-amoxiclav prescriptions between males and females (Table 1).

### 2.2. Urine Culture

A urine sample was obtained during 1286 or 50% of the index consultations. Samples were submitted from 135 (48%) males and 1150 (51%) females (Table 1). A lower percentage of the urine samples from male patients showed a positive culture compared to the female urine samples (36% vs. 45%). The percentage breakdown of species identified as positive culture results for males and females is shown in Table 1.

**Figure 2:**
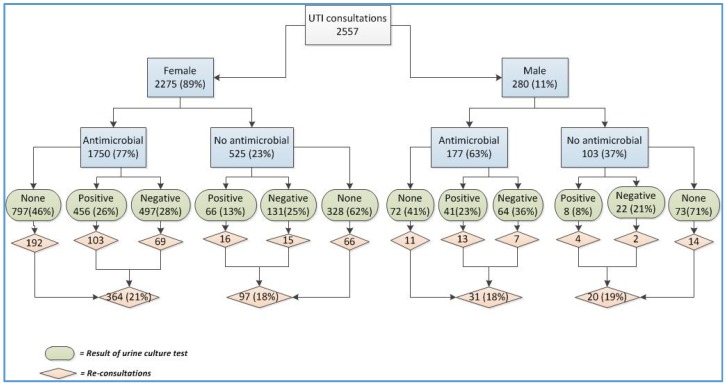
Flow chart of the reconsultations according to gender, antimicrobial treatment, and culture results.

Of the 49 male patients who had a positive culture, 41 received an antimicrobial, which was generally nitrofurantoin (66%). Of the patients who received an antimicrobial, 13 had a subsequent reconsultation, of whom one may have been related to the non-susceptibility of the organism to the antimicrobial prescribed (i.e., nitrofurantoin was prescribed for *Proteus* spp.). Of the 522 females with positive cultures, 456 received an antimicrobial, of whom 76% received nitrofurantoin. Of the subsequent 129 reconsultations, seven may have been due to the non-susceptibility of the organism to the prescribed antimicrobials (one *Proteus* spp. where nitrofurantoin was prescribed, six organisms identified with resistance against the antimicrobial that was prescribed) (Figure 2 flow chart).

### 2.3. Reconsultation

**Figure 3:**
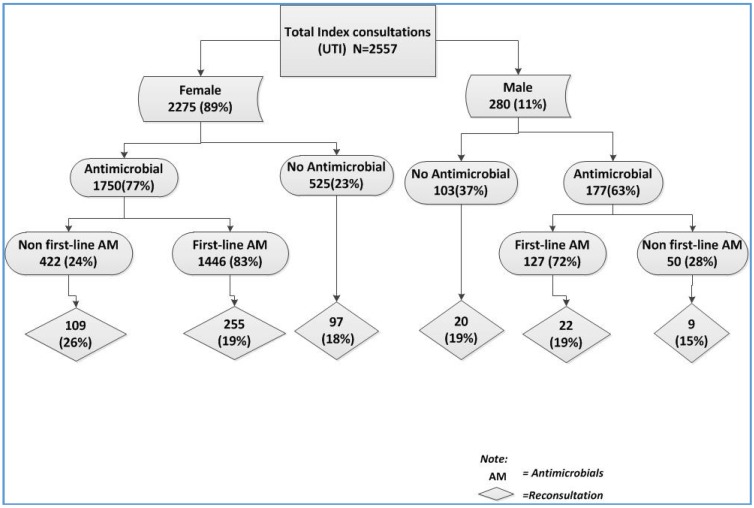
Flow chart of the reconsultations according to gender and first-line treatment of antimicrobials.

A logistic regression analysis with outcome reconsultation showed females to have a higher occurrence of reconsultation compared to males after correction for confounding factors (age, medical card status, and whether an antimicrobial was prescribed) (Table 2). However, no significant differences were observed in reconsultation between males and females when an antimicrobial was the first-line (nitrofurantoin) (Figure 3 flow chart). When an antimicrobial was prescribed empirically and was not nitrofurantoin, the odds of reconsultation were higher (OR 1.56, 95% CI (1.1–2.0)). There was no difference in reconsultations when quinolones were prescribed empirically, but the odds of reconsultation were significantly higher when trimethoprim was prescribed (OR 2.0 95% CI (1.3–3.1)) (not shown in Table 2). However, neither the gender nor the antimicrobial prescribing was significant in a model in which culture test results were included. In the subset of patients in which a urine culture was performed, the positive culture test was associated with reconsultation (OR = 1.8, 95% CI 1.3–2.5) (not shown in Table 2).

## 3. Discussion

A urine sample was only available for approximately 50% of the patients. Even though there is no indication that patients without culture results had better or worse outcomes, it is unclear as to why no urine sample was obtained from the other half of the UTI patients. Studies indicate that empiric antimicrobial treatment without urine culture is appropriate for uncomplicated UTI in primary care, however for males, a urine culture rather than empiric treatment is recommended [5,6,8]. Conversely, the Scottish Intercollegiate Guidelines Network (SIGN) recommends both urine culture and empiric treatment for all male with symptoms of UTI [10]. 

Males were less often prescribed an antimicrobial empirically, but if an antimicrobial was prescribed, the duration of treatment was generally longer and the antimicrobial prescribed was more likely to be a fluoroquinolone. Cultures from males were less likely to be positive and re-consultations were less common. However, for both males and females, reconsultation was less common when nitrofurantoin was prescribed compared with prescription of any other antimicrobial or compared with no antimicrobial prescription. A study conducted in Germany also supports following local (first-line) antimicrobial guidelines for both males and females when prescribing empirically [7]. 

Conventional practice with respect to management of UTI has emphasized empiric antimicrobial prescribing at the first consultation. This is challenged by an emerging body of evidence that shows that symptomatic treatment and delayed antimicrobial use for UTI in females is safe and allows for spontaneous resolution in a high proportion of cases. Our study points to differences between males and females presenting with features suggestive of UTI with respect to the likelihood of a positive culture, management decisions, and frequency of reconsultation. Given these differences, it is important that studies reassess conventional practices with respect to UTI and address both male and female patients independently. 

The data used in this study were obtained through a remote electronic download from the general practice patient management software through the Irish Primary Care Research Network (IPCRN), and are therefore reliable and complete. However, even though all the data on urinary tract infection coded as U71 consultations is captured, coding depends on the General Practitioners (GPs). GPs may have been less inclined to code for male consultations as it was not explicitly requested to include females and males. Even though this does not impact the outcome of reconsultation, the number of females may be over represented. Another limitation of the study was that no detailed information was available from any other UTI related consultation; only “suspected UTI” (U71) was recorded. If the GP considered a male UTI as complicated or if the consultation was not recorded as U71, no data was downloaded. Also, whether patients were referred to a urologist or other specialist for further investigation and management was not recorded. Similarly, no symptoms nor any further diagnostic information, such as dipstick results or other predictive indicators, were recorded in the GP patient management software as this was an observational study of patient records.

## 4. Materials and Methods

This is a secondary data analysis of the Supporting the Improvement and Management of Prescribing for Urinary tract infection (SIMPle) study [11]. The study was conducted in 30 general practices in the west of Ireland, for a period of 15 months, to improve the quality of antimicrobial prescribing for UTIs in general practice. GPs were requested to code all consultations (U71) with patients with suspected UTI. Remote electronic data collection was initiated from the practice’s patient management software through the Irish Primary Care Research Network (IPCRN) [12], a national research network of general practices. At the start of the study, all practices received a workshop on consultation coding after which practices were allocated to intervention (arm A and B) and control groups (arm C). The intervention arm A received a workshop on appropriate prescribing for UTI supported by practice specific audit reports and the intervention arm B received a workshop on delayed prescribing including the intervention package in arm A. There was no specific intervention delivered to intervention arms in relation to the management of UTI in males.

From June 2013 to August 2014, data was collected on all adult patients with suspected UTI in the 30 general practices. No specific guidance was given regarding the diagnosis of UTI, in order to interfere as little as possible with daily clinical care. GPs were encouraged to submit a urine sample from all suspected UTI patients. The IPCRN provided data on patients’ age, gender, medical card status, consultation date, type of prescription, and treatment (Anatomical Therapeutic Chemical (ATC) code). A medical card provides the holder with free healthcare and medication. Entitlement to a medical card is based on income and age—97% of those aged 70 and older and about one-third of the population under 70 have a medical card [13].

Ethical approval for the SIMPle study was obtained from the Irish College of General Practitioners (ICGP).

### 4.1. UTI and Urine Culture

A UTI episode was defined as a clinical consultation for which a U71 consultation code was entered. For each of these patients, information on previous and subsequent consultations as well as antimicrobial therapy was extracted. Urine culture results and antimicrobial susceptibility profiles of isolates were also extracted. Standard microbiological methods were used for the detection and identification of pathogens. UTI was considered laboratory confirmed when bacterial growth of >10^5^ pure culture/mL was detected. Susceptibility to amoxicillin, co-amoxyclav, trimethoprim, ciprofloxacin, nitrofurantoin, and cefpodoxime was performed by EUCAST disc diffusion and interpreted according to EUCAST guidelines [14].

### 4.2. Reconsultation

The first UTI episode was classified as the index consultation and reconsultation was identified as a further consultation within 30 days of the first visit for the same reason (UTI).

### 4.3. Empirical Prescribing

At the time of the study, nitrofurantoin and trimethoprim were recommended first-line treatment for males and females according to national guidelines. However, the guidelines recommend the use of these first-line antimicrobials when resistance levels among common pathogens is below a threshold of 20% [15]. As trimethoprim resistance in *E. coli* in this region has been greater than 20% for some years, this means that effectively nitrofurantoin is the only first-line treatment recommended [16]. According to the current national guidelines, the first-line recommended agent is used for different durations; seven days in males and three days in females. Fluoroquinolones (such as ciprofloxacin) were considered a reserve antimicrobial and should only be prescribed after culture results and susceptibility is known [3]. For the purpose of analysis, the dose dispensed (for instance 12 tablets four times daily equals a duration of 3 days) was considered as the duration of the treatment according to the recommended daily dose.

### 4.4. Statistical Analysis

The presented secondary analyses were performed with data generated during the SIMPle study. An overview of the patients is presented according to gender. General demographic profiles of the patients in relation to antimicrobial prescribing are presented in percentages as well as means and medians of age. A binary logistic regression model was applied and results were presented as an odds ratio (OR) and associated 95% confidence interval (CI). Interactions were tested and omitted from the models if they were not significant. Overall statistical analysis was performed with IBM SPSS Statistics for |Windows Version 21.0, (IBM Corporation released 2012, Armonk, NY, USA). Flow diagrams and figures presented in the paper were produced using Microsoft Visio 2010 and Microsoft Excel 2010 (Microsoft Corporation, Redmond, WA, USA).

## 5. Conclusions

After allowing for differences in prescribing (first-line, non first-line, or no antimicrobial), no significance differences were observed in reconsultation among male and female UTI patients. When prescribing antimicrobial treatment for UTI empirically, first-line treatment (nitrofurantoin) should be the preferred choice for both males and females. 

## Figures and Tables

**Table 1:** Univariate comparisons of the index consultation between males and females.

Characteristics	Male		Female		*p*-Value
	*N*	%	*N*	%	
Index consultation	280	11.0	2275	89.0	
Reconsultation	51	18.2	461	20.3	ns
Age in years (mean and SD)	63.6	17.8	52.4	20.9	**<0.001**
17–25	8	2.9	275	12.0	
26–50	52	18.6	802	35.3	
50–75	134	47.9	814	35.8	
>75	86	30.7	384	16.9	
Medical card	194	69.3	1372	60.3	**0.004**
Arms					
Intervention arm A	100	35.7	698	30.7	ns
Intervention arm B	89	31.8	827	36.4	
Control arm	91	32.5	750	33.0	
Antimicrobial prescribed	177	63.2	1750	76.9	**<0.001**
Types of antimicrobial prescribed					
Nitrofurantoin	116	41.4	1328	58.4	**<0.001**
Trimethoprim	11	3.9	118	5.2	ns
Fluoroquinolone	31	11.1	71	3.1	**<0.001**
Co-amoxiclav	14	5.0	140	6.2	ns
Amoxicillin	3	1.1	43	1.9	ns
Duration of treatment (median, min-max) where antimicrobial was prescribed	7	1–14	3	1–28	**<0.001**
Duration of treatment (days) for types of antimicrobial prescribed (median, min-max)					
Nitrofurantoin	7	1–10	3	1–28	**<0.001**
Trimethoprim	5	1–14	5	1–14	**0.002**
Fluoroquinolone	5	1–10	5	1–10	ns
Co-amoxiclav	7	1–7	7	1–15	ns
Urine sample obtained	135	48.2	1150	50.5	ns
Positive culture	49	36.3	522	45.4	**0.04**
Resistance to any tested antimicrobial	33	73.3	268	56.3	**0.02**
Types of bacteria (among + ve culture)					
*E. coli*/*coliform*	40	14.3	442	19.4	**0.03**
*Proteus* spp.	2	3.3	17	2.4	ns
*Staphylococcus*	0		28		
Other	7		35		

ns = not significant. The significant result with *p* value < 0.05 is highlighted in bold.

**Table 2:** Logistic regression of re-consultation for UTI.

Characteristics	OR	95% CI	*p*-Value
Gender			
Male	reference		
Female	1.4	1.0–1.9	**0.04**
Age	1.02	1.0–1.02	**<0.001**
Medical card			
No	reference		
Yes	1.7	1.3–2.1	**<0.001**
Prescribed antimicrobials			
No	reference		
Yes	1.2	0.96–1.5	0.1
Intervention			
Control (arm C)	reference		
Intervention (arm A and B)	0.6	0.5–0.8	**<0.001**
